# Supplementary material for: Anti-inflammatory cytokine profile and Jarisch-Herxheimer reaction in Leptospirosis patients: A prospective case-series study in New Caledonia
Source: PLoS Negl Trop Dis. 2025 Sep 23;19(9):e0013189. doi: 10.1371/journal.pntd.0013189 (PMC12494262; doi:10.1371/journal.pntd.0013189)

**S1 Fig. Flow of the study protocol for inclusion and data and sample collection, LEPJAR-NC study, New Caledonia, 2021-2024**


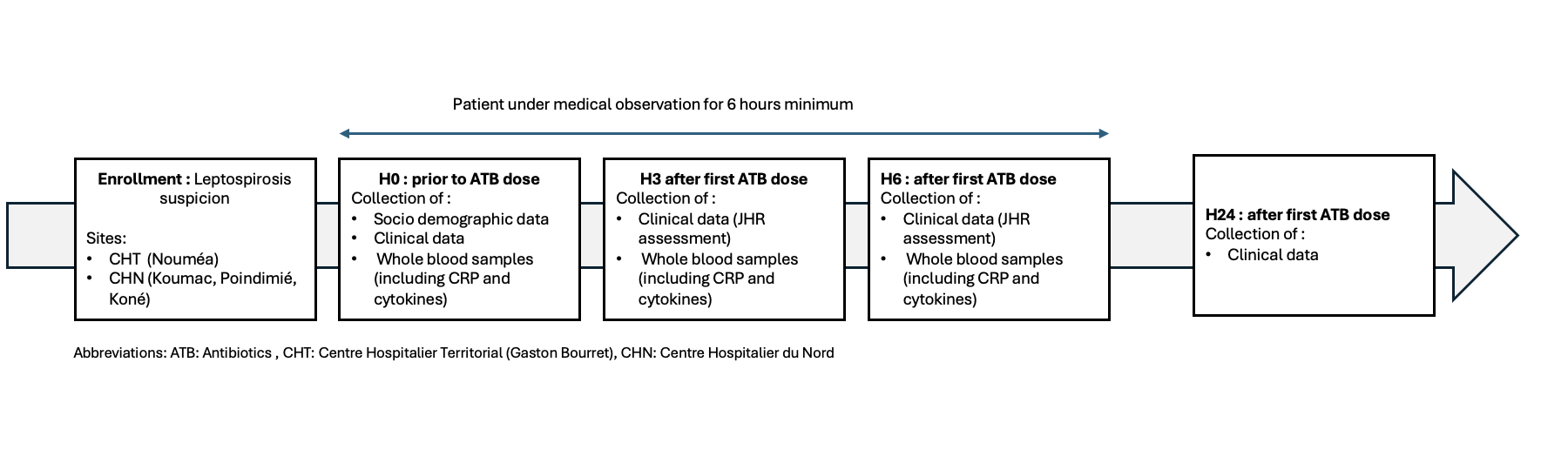

Supplement: S1 Fig — (DOCX) [file pntd.0013189.s001.docx]
