# Supplementary material for: Anti-inflammatory cytokine profile and Jarisch-Herxheimer reaction in Leptospirosis patients: A prospective case-series study in New Caledonia
Source: PLoS Negl Trop Dis. 2025 Sep 23;19(9):e0013189. doi: 10.1371/journal.pntd.0013189 (PMC12494262; doi:10.1371/journal.pntd.0013189)
Supplement: S1 Table — (DOCX) [file pntd.0013189.s002.docx]

**S1 Table. Description of the leptospirosis treatment of the enrolled patients, LEPJAR-NC Study, New Caledonia, 2021-2024**

|  | **Total**  **N** | **CHT**  **n (%)** | **Koumac**  **n (%)** | **Kone**  **n (%)** | **Poindimie**  **n (%)** |
| --- | --- | --- | --- | --- | --- |
| **Amox/ full dose** | 3 | 3 (100) | 0 | 0 | 0 |
| **Amox/progressive/Hydrocortisone** | 33 | 0 | 33(100) | 0 | 0 |
| **3GC full dose** | 28 | 19 (67.86) | 0 | 6 (21.43) | 3 (10.71) |
| **3GC/full dose/Methylprednisone** | 5 | 0 | 0 | 0 | 5(100) |
| **3GC + nitromidazoles/full dose** | 1 | 0 | 0 | 0 | 1(100) |
| **3GC +aminoside/full dose** | 10 | 7(70.00) | 0 | 3(30.00) | 0 |
| **Missing** | 1 | 0 | 0 | 1(100) | 0 |

Acronyms: Amox: Amoxicillin, 3GC: 3^rd^ generation cephalosporin, CHT: Centre Hospitalier Territorial (Gaston Bourret)
