# Supplementary material for: Anti-inflammatory cytokine profile and Jarisch-Herxheimer reaction in Leptospirosis patients: A prospective case-series study in New Caledonia
Source: PLoS Negl Trop Dis. 2025 Sep 23;19(9):e0013189. doi: 10.1371/journal.pntd.0013189 (PMC12494262; doi:10.1371/journal.pntd.0013189)
Supplement: S3 Table — (DOCX) [file pntd.0013189.s004.docx]

**S3 Table. Description of the socio-economic factors of enrolled patients according to the leptospiral genotype, LEPJAR-NC Study, New Caledonia, 2021-2024**

|  | **B1 GT *** | **I1 GT *** | **I2 GT *** | **I5 GT *** | **Missing GT*** | **p-value**** |
| --- | --- | --- | --- | --- | --- | --- |
|  | **n/N (%) or** | **n/N (%) or** | **n/N (%) or** | **n/N (%) or** | **n/N (%) or** |  |
|  | **median [IQR]** | **median [IQR]** | **median [IQR]** | **median [IQR]** | **median [IQR]** |  |
| **Hospital**  CHT | 3 (37.50%) | 20 (50%) | 0 (0%) | 1 (11.11%) | 5 (35.71%) | **0.00231** |
| Koumac | 5 (62.50%) | 9 (22.50%) | 5 (50%) | 6 (66.67%) | 8 (57.14%) |  |
| Kone | 0 (0%) | 8 (20%) | 1 (10%) | 1 (11.11%) | 0 (0%) |  |
| Poindimie | 0 (0%) | 3 (7.50%) | 4 (40%) | 1 (11.11%) | 1 (7.14%) |  |
| **Age (year)** | 50 [44.75;64.25] | 44.50 [34;59] | 48 [43;54.75] | 35 [27;52] | 31 [25;38] | 0.29972 |
| **Sexe** Female | 0 (0%) | 13 (32.50%) | 4 (40%) | 2 (22.22%) | 3 (21.43%) | 0.22032 |
| Male | 8 (100%) | 27 (67.50%) | 6 (60%) | 7 (77.78%) | 11 (78.57%) |  |
| **Community**  Melanesians | 3 (37.50%) | 27 (67.50%) | 8 (80%) | 7 (77.78%) | 10 (71.43%) | 0.14454 |
| Europeans | 3 (37.50%) | 1 (2.50%) | 1 (10%) | 1 (11.11%) | 1 (7.14%) |  |
| Other Communities † | 1 (12.50%) | 3 (7.50%) | 0 (0%) | 0 (0%) | 2 (14.29%) |  |
| Missing/ not declared | 1 (12.50%) | 9 (22.50%) | 1 (10%) | 1 (11.11%) | 1 (7.14%) |  |
| **Comorbidities**  No | 4 (66.67%) | 26 (78.79%) | 7 (100%) | 7 (100%) | 13 (100%) | 0.24988 |
| Yes | 2 (33.33%) | 7 (21.21%) | 0 (0%) | 0 (0%) | 0 (0%) |  |
| **Occupation** Farmer | 3 (50%) | 7 (29.17%) | 6 (100%) | 3 (60%) | 5 (41.67%) | 0.06817 |
| Out of work | 1 (16.67%) | 9 (37.50%) | 0 (0%) | 0 (0%) | 3 (25%) |  |
| Workman | 1 (16.67%) | 7 (29.17%) | 0 (0%) | 2 (40%) | 1 (8.33%) |  |
| Self-employed | 1 (16.67%) | 0 (0%) | 0 (0%) | 0 (0%) | 1 (8.33%) |  |
| Student | 0 (0%) | 0 (0%) | 0 (0%) | 0 (0%) | 2 (16.67%) |  |
| Manager | 0 (0%) | 1 (4.17%) | 0 (0%) | 0 (0%) | 0 (0%) |  |
| **Animals linked activities** Yes | 6 (85.71%) | 27 (75%) | 9 (90%) | 7 (87.50%) | 13 (92.86%) | 0.81149 |
| No | 1 (14.29%) | 9 (25%) | 1 (10%) | 1 (12.50%) | 1 (7.14%) |  |
| **Field occupation**  Yes | 4 (57.14%) | 23 (63.89%) | 7 (70%) | 6 (75%) | 10 (71.43%) | 0.90022 |
| No | 3 (42.86%) | 13 (36.11%) | 3 (30%) | 2 (25%) | 4 (28.57%) |  |
| **Freshwater related activities** Yes | 4 (57.14%) | 18 (50%) | 7 (70%) | 8 (100%) | 10 (71.43%) | **0.04774** |
| No | 3 (42.86%) | 18 (50%) | 3 (30%) | 0 (0%) | 4 (28.57%) |  |

Acronyms: CHT: Centre Hospitalier Territorial, GT: Genotype, IQR: Inter-quartile Range

* Genotype-serovars correspondances:

genotype I1: *Leptospira interrogans* serovar Icterohaemorragiae,

genotype I2 : *Leptospira* *interrogans* serovar Australis,

genotype I5 : *Leptospira* *interrogans* serovar Pyrogenes,

genotype B1: *Leptospira borgpeterseni* serovar Ballum

** Fisher’s exact test/ Kruskal-Wallis Test (Non-Parametric ANOVA) to assess differences between the genotypes

† Other communities include Asian, Polynesian, Indonesian, Vietnamese, and people from Vanuatu.
