# Supplementary material for: Anti-inflammatory cytokine profile and Jarisch-Herxheimer reaction in Leptospirosis patients: A prospective case-series study in New Caledonia
Source: PLoS Negl Trop Dis. 2025 Sep 23;19(9):e0013189. doi: 10.1371/journal.pntd.0013189 (PMC12494262; doi:10.1371/journal.pntd.0013189)
Supplement: S4 Table — (DOCX) [file pntd.0013189.s005.docx]

**S4 Table.** **Description of biological data of enrolled patients according to leptospiral genotype, LEPJAR-NC Study, New Caledonia, 2021-2024**

|  | **B1 GT*** | **I1 GT*** | **I2 GT*** | **I5 GT*** | **Missing GT*** | **p-value**** |
| --- | --- | --- | --- | --- | --- | --- |
|  | **n/N (%) or** | **n/N (%) or** | **n/N (%) or** | **n/N (%) or** | **n/N (%) or** |  |
|  | **median [IQR]** | **median [IQR]** | **median [IQR]** | **median [IQR]** | **median [IQR]** |  |
| **Leptospiral load** (Leptospires/mL) | 267  [146;2024.50] | 3165  [130;15437] | 234  [100 ;668] | 1077  [422 ;25817] | 88  [8 ;1265] | 0.06254 |
| **Leucocytes** (/µl) | 9,200 [7092.50;11725] | 12,660  [8865 ;14890] | 12,660 [10000;13100] | 12,600 [8700;13200] | 9,600 [7550;11580] | 0.30223 |
| **Lymphocytes** (/µl) | 450  [325;900] | 560  [420;800] | 760  [500;1000] | 500  [400;700] | 700  [500;1200] | 0.64406 |
| **Neutrophiles** (/µl) | 6700  [4980;8225] | 11550 [7605;13652.50] | 11200 [9300;12400] | 11600 [7400;12000] | 8800 [7000;10950] | 0.09873 |
| **Monocytes** (/µl) | 645  [360;880] | 510  [360;740] | 500  [287.50;650] | 550  [282.50;600] | 400  [200;740] | 0.74996 |
| **Thrombocytes** (/µl) | 130,000 [110750;168250] | 41,000 [25250;84250] | 129,000 [97000;133000] | 105,000 [30000;120000] | 71,500 [63000;124250] | **0.00019** |
| **Hematocrit (%)** | 43.30  [38.50;45.50] | 38  [35.70;41] | 39.50  [36;42] | 39.50  [36.92;42.50] | 37  [35;41] | 0.43394 |
| **Hemoblobin** (g/dl) | 14.80  [12.65;16] | 13  [12;14] | 13.40  [12.50;15] | 14  [13.53;14.25] | 14  [13;16] | 0.46403 |
| **Urea (mmol/L)** | 6.95  [5;9.55] | 12  [7;21] | 8  [6;15] | 6  [5;7] | 5  [5;12.50] | 0.07058 |
| **Creatinine** (µmol/L), | 111  [99;124] | 191  [121;376] | 107  [83;203] | 113  [102;129] | 100  [87;229] | **0.03925** |
| **ASAT** (UI/L), | 71  [61;157] | 110  [53;232] | 37  [33;46] | 42  [34;67] | 68  [49;104] | **0.01248** |
| **ALAT** (UI/L), | 71  [45;156.50] | 57  [31;109] | 36  [26;38] | 56  [23;66] | 68  [52;107] | 0.16488 |
| **Gamma-GT** (UI/L), | 321.50  [247;387] | 67  [29;100] | 21  [21;21] | 33  [33;33] | 76  [51;105] | 0.05964 |
| **Bilirubin** (µmol/L), | 23.70  [22;24.40] | 70.05  [22.50;168] | 16.50  [14;23.50] | 17.50  [14;23] | 29.10  [20;63] | **0.00762** |
| **CRP** (mg/L), | 208  [175;226] | 255  [162;322] | 236  [200;288] | 235  [136;314] | 197.50  [181;260] | 0.53140 |
| **TNF** (pg/mL) | 72  [45;139] | 61  [27;100] | 34  [21;42] | 70 [33;78] | 13  [0;28] | 0.35632 |
| **IL-10** (pg/mL) | 2213  [1370;2948] | 2491  [953;3444] | 548  [290;1334] | 1894  [996;3277] | 403  [82;1022] | **0.03679** |
| **IL-1ß** (pg/mL) | 0  [0;19] | 0  [0;0] | 0  [0;3] | 3  [0;40] | 0  [0;0] | **0.00364** |
| **IL-6** (pg/mL) | 0  [0;0] | 0  [0;129] | 0  [0;0] | 19.50  [0;72] | 0  [0;0] | 0.21693 |

Acronyms: GT: Genotype, IQR: Inter-quartile Range

* Genotype-serovars correspondances:

genotype I1: *Leptospira interrogans* serovar Icterohaemorragiae,

genotype I2 : *Leptospira* *interrogans* serovar Australis,

genotype I5 : *Leptospira* *interrogans* serovar Pyrogenes,

genotype B1: *Leptospira borgpeterseni* serovar Ballum

** Fisher’s exact test/ Kruskal-Wallis Test (Non-Parametric ANOVA) to assess differences between genotypes.

† Normal blood cells value in adult healthy population, source CHT, Nouméa: Leucocytes: 4 – 10 G/L, Lymphocytes: 1 - 5 G/L, Neutrophiles: 2 - 7.5 G/L, Monocytes: 0.2 – 1 G/L, Thrombocytes 150 - 400 G/L (150 000-400 000/µl), Hematocrit: women: 38-47% and men 40-54%, Hemoglobin: women 12-16 g/dl and men 13-17 g/dl;

‡ Biochemical parameter in adult healthy population, source: CHT, Nouméa: urea: 2.5 to 9.2 mmol/L (depending on age and sex), creatinine: 49 -104 µmol/L (depending on age and sex), ASAT: 5-34UI/L, ALAT: 0-55 UI/L, G-GT: <38 for women and <55 for men, bilirubin total: 5.1-20.5 µmol/L; CRP <5 mg/l

§ Cytokine levels in healthy humans [26]; TNF <30 pg/mL, IL-10 <17 pg/mL, IL-1ß not detected, IL-6<15 pg/mL For each parameter, n represents the total number of patients tested. Values in bold are outside the norm, or, for p values, indicate a significant difference between sites.
