# Supplementary material for: Anti-inflammatory cytokine profile and Jarisch-Herxheimer reaction in Leptospirosis patients: A prospective case-series study in New Caledonia
Source: PLoS Negl Trop Dis. 2025 Sep 23;19(9):e0013189. doi: 10.1371/journal.pntd.0013189 (PMC12494262; doi:10.1371/journal.pntd.0013189)
Supplement: Attachment — Submitted filename: Answers to Reviewers_JHR_25-08-19.docx [file pntd.0013189.s006.docx]

**Point-by-point response to the reviewers.**

We would like to thank the reviewers for their interesting comments, questions and suggestions. We hope that the provided answers and modifications have improved the manuscript.

For clarity, we first answered all reviewer1's questions, then all reviewer2's questions. The reviewers' comments are in black, as are **their questions** written in bold. Our comments and answers are in blue, and the modifications in the manuscript are underlined.

References in yellow correspond to the one in the manuscript. Additional references are appended below.

**Reviewer 1’s Questions and** answers:

**Methods**

Reviewer #1: JHR clinical. The new JHR clinical scoring system is an important step forward and seems to have been validated by the various lab results presented in the paper. It would be helpful to understand the JHR score criteria more clearly. For example, **how is fever defined (above what temperature)? How much of change in O2 saturation is considered a “decrease”? Do these changes need to happen within a particular time (eg 6 hours?) after treatment? How does the JHR score compare to JHR definitions used by others for syphilis or leptospirosis?** Defining these criteria more clearly would be helpful for future studies by others.

Answer: Thank you, we now added the missing information (highlighted in yellow) in the material and methods section; line 236: “JHR was defined as follows during the first 6 hours of treatment: onset or recurrence of fever (defined as a temperature ≥ 38°C) or onset of chills or myalgia were considered as 2 major criteria, while increased heart rate above 20%, increased respiratory rate above 20%, and hypoxaemia (defined as a O2 saturation below 95% were considered as 3 minor criteria (Table 1). Blood pressure variation was not included because it could have been confused with a worsening of leptospirosis. The criteria evolution was defined compared to the baseline assessment (H0) before antibiotherapy and if happening within the first 6 hours after treatment initiation.

The Figure 2 (now renamed Table 1) has been changed accordingly.

**Table 1**: New JHR score: major and minor criteria according to the clinical status, LEPJAR-NC study, New Caledonia, 2021-2024

| **Major criteria** | **Minor Criteria** |
| --- | --- |
| Onset or recurrence of fever (ear temperature ≥ 38°C | Heart rate increase (increase of more or equal than 20% between the start of treatment and hour 6) |
| Onset or recurrence of chills or myalgias (reported by patients themselves) | Respiratory rate increase (increase of more or equal than 20% between the start of treatment and hour 6) |
|  | O_2_ saturation decrease (O2 saturation decreasing under 95%) |

# Answer about JHR score for other *Leptospira* studies or syphilis:

# A systematic review of Guerrier in 2013 about JHR and leptospirosis stated that *” the methods used to monitor JHR were not described in enough detail to reach predefined criteria and determine whether there was a link between JHR occurrence and organ failure or death”* [1]. Since 2013 in the *Leptospira* field, there was to the best of our knowledge only one large retrospective clinical study in Japan [2] which defined *“JHR to be a patient that had documented new shaking chills, a lower blood pressure (systolic blood pressure of <90 mmHg), or a spiking body temperature higher than that recorded before administration of antibiotics.”*

# Recent randomized clinical trials on patients with syphilis either to better define JHR among 700 neurosyphilis patients without HIV [3] or to test different treatments with Benzathine Penicillin G [4] showed that 10 to 20 % of patients, may also have a JHR, defined by one or more symptoms among a long list, including the frequently noticed fever, rash, myalgias, headache, tachypnoea, tachycardia, nausea, hypotension, hyperventilation and exacerbation of skin lesions occurring, within 2 to 12 hours of initial treatment. Kapila and Schartz recently attempted to classify JHR occurring in neurosyphilis in patients with HIV in 3 stages according to severity, primarily based on the above symptoms, with self-limited stage 1, moderate stage 2 with hypotension requiring pressors, and with a stage 3 involving serious complications such as circulatory shock, myocarditis, acute respiratory distress, seizures, meningitidis, strokes and death [3]. However, this study was based on literature and one case report and did not discuss the confounding factors between severity and JHR.

Therefore, to the best of our knowledge, in the field of syphilis, or in other studies on leptospirosis, a precise JHR definition or scoring is missing. That is why we tried in the present study to better define JHR with a score that could be used in subsequent clinical studies. We added a sentence in the introduction section line 158: One issue in the fields of leptospirosis and syphilis is the lack of a precise definition or scoring system for JHR, that would allow for easier comparison between studies [1-5].

The absence of measurable IL-1β is remarkable. **Were positive controls included to make sure the assay was working?** **What is the sensitivity of the assay kit used to measure this cytokine?** IL-1β is typically present in picogram-range concentrations and some detection methods may lack sensitivity. The authors should acknowledge IL-1β usually peaks early during an infection and may be compartmentalized during infection. Systemic IL-1β is rapidly cleared even as downstream cytokines like IL-6 and TNF remain elevated. IL-10 can act to suppress IL-1β production.

Answer: Yes, each DuoSet ELISA kit (R&D Systems) included a Human IL-1β standard protein, which was used to generate a standard curve (concentrations: 3.91, 7.81, 15.62, 31.25, 62.5, 125, and 250 pg/mL). These standards were run in duplicate for every experiment (on each 96-well plate) and served both to establish the standard curve and as positive controls. Negative controls were also included to ensure assay specificity. This procedure was followed consistently for each experiment to verify the accuracy and reproducibility of the measurements. According to the manufacturer, the validated detection range for the Human IL-1β assay is from 3.91 to 250 pg/mL. Only values within this quantifiable range were considered for analysis.

To confirm the quasi-absence of IL-1ß, we tested our human IL-1β DuoSet kit (from R&D Systems) on whole blood from a healthy volunteer infected with leptospires for 24 hours. We compared this to stimulation with purified agonists from InvivoGen (ultrapure LPS at 10 ng/mL and Pam3cys SK4 at 100 ng/mL). As shown in the graph below, the TLR4 agonist LPS induced a fair amount of IL-1β secretion, as did the TLR2 agonist Pam2cys to a lesser extent. However, no IL-1β was obtained with infection with 5.10⁵ leptospires, which exceeds the bacterial loads measured in the blood of patients. These data were not included in the manuscript, but we added in the mat/met section line 207: Quality controls have been performed according to the manufacturer recommendation to ensure the validity of the laboratory results in human sera.

However, we were also surprised by the lack of IL-1β production in the blood of patients with leptospirosis. We compared with different studies about leptospirosis patients. the following paragraph was added in the discussion line 552: In comparison, a 2015 study measured IL-1β levels in the sera of 47 patients with acute leptospirosis at a mean of 40 pg/mL. However, this level was not significantly different from that of healthy controls [31]. Similar IL-1β levels were found in a 2013 Brazilian study, though leptospirosis patients exhibited higher levels of IL-1β than controls. However, there was no difference between mild and severe patients [34]. These results differ greatly from those of studies on sepsis. For example, in a 2016 study of patients with acute cystitis, the mean IL-1β level was 500 pg/mL, whereas control groups exhibited undetectable levels of IL-1β [35].

Results

Reviewer #1: Clinical evolution. Of the 81 patients, apparently only 59 patients had JHR information recorded on the case report form. Please confirm whether the movement to ICU occurred during the observation period after treatment. **Is it possible to correlate any of the time=0 laboratory results with subsequent movement to ICU?** Movement to ICU apparently interfered with JHR information collection. **At what timepoints after treatment did movement to ICU occur in the 11 patients?**

Answer: thank you for this comment. However, information about time of movement to ICU and the treatment time was not collected in the context of the study. We added a sentence in the result section line 394: No additional information concerning the transfer to ICU (time, clinical and biological assessment or care) was collected.

We also added a sentence in the discussion section line 642: This study has several limitations that reduce its scope and significance. First, the delay between onset of first leptospirosis symptoms and antibiotic treatment was not recorded in the present study. It would have been an important parameter to consider confirming the above hypothesis.

**Is there any indication that movement to ICU was associated with any particular type of treatment, more severe JHR, or leptospiremia level?** Related question: **What was the range of JHR severity? Did movement to ICU or JHR occur more or less frequently in the patients who received a particular treatment modality such as escalating doses of amoxicillin and/or steroids?**

Answer: the movement of ICU was associated to severity of the clinical, biological presentation of the patients at admission, and there was no link with the type of treatment. Transfer from the North may also be motivated by minimal surveillance capacity. Any patient transferred from the North health facilities was directly admitted to the CHT ICU. We added this information in the result section line 390. As we have already specified line 613 the number of patients and the lack of a unique treatment regimen did not allow us to look for any association between the treatment and ICU/JHR.

**In other words, is there any indication of clinical benefit from the escalating doses of amoxicillin and/or steroid protocols**? Answering this question could be a subject of a subsequent clinical trial.

Answer: we agree that a subsequent randomized clinical trial should be set up to answer this important question. Meanwhile, as mentioned in the discussion line 546, ongoing studies using human blood from healthy volunteers and infected mice with *Leptospira* should give hints about the mechanism of JHR, but also determine whether the escalating protocols with ß-lactams antibiotics and/or steroids can induce less inflammation compared to full-dose protocols.

Is one possible explanation for the lower liver and renal parameters in the JHR patients is that they were treated earlier in the course of infection than the patients without JHR. In other words, the patients without JHR had already cleared bacteremia because they were at a later stage of leptospirosis.

Answer: yes, we agree, and this hypothesis was highlighted in the discussion section line 608.

**Were any data collected from patients regarding the duration of time from onset of symptoms to treatment?**

Answer: We acknowledge that that it is a real missed opportunity that the time between onset of symptoms and treatment was not collected. This missing information constitutes a bias of our study. We have added a sentence in the discussion about the limitations of our study line 613 First, the delay between onset of first leptospirosis symptoms and antibiotic treatment was not recorded in the present study. It would have been an important parameter to consider confirming the above hypothesis”.

**Conclusions**

Reviewer #1: The authors should acknowledge that there are alternative explanations for the elevated IL-10 levels. IL-10 is an anti-inflammatory cytokine that can become elevated in a compensatory manner during severe infection. In the 2004 study of patients with septic shock by Monneret et al. (PMID: 15388260), IL-10 levels were correlated with severity and survival. **In this JHR study, were IL-10 levels associated with more severe infection? How do the IL-10 levels observed in this JHR study compare to what has been observed in other studies of leptospirosis or other infectious diseases?** The authors are encouraged to explore the nexus between IL-10 immunosuppression, high levels of leptospiremia and JHR. The study results show higher IL-10 levels and higher levels of leptospiremia in the JHR patients than non-JHR patients. Based on these results, it seems plausible that IL-10 immunosuppression contributed to leptospiremia and that leptospiremia contributed to JHR.

Answer: Yes, we agree that the reviewer’s hypothesis would make sense.

In animal models, the anti-inflammatory IL-10 cytokine has been noticed to be associated with severity. Interesting studies suggest that IL-10 antagonize the innate defense and is deleterious for the host favoring renal colonization in mice [6], or that IL-10 absence is protective for the hamster with reduced bacterial loads [7]. We recently showed that lethally infected mice, mimicking severe human leptospirosis also produced large amounts of IL-10, and notably low levels of inflammatory cytokines [8]. IL-10 secretion in leptospirosis patients’ sera have been recorded in numerous studies. The levels observed in our study are consistent with a Sri-Lankan study showing IL-10 production in leptospirosis patients in a range of 26 to 6176 pg/mL, or with a Brazilian 2013 study [9] showing that IL-10 and IL-6, among other cytokines, increased in severe lethal leptospirosis compared to mild cases. In contrast, another recent descriptive Sri-Lanka study found IL-10’s levels below 300 pg/ml. A descriptive recent Iranian study which also showed that IL-10 levels significantly increased in patients with severe disease and were even higher in fatal cases, mentioned very low levels of IL-10, in the range of 10 to 20 pg/ml [10], as in a study conducted in Indonesia [11]. In this line, a systematic review about leptospirosis patients showed different mean levels of IL-10 and TNF between several studies, and concluded about “*equivocal findings on the association between IL-1beta, TNF-alpha and IL-10/TNF-alpha ratio and disease severity*”[12].

We may speculate that this variability in cytokine levels could be due to infection with different serovars of *Leptospira,* as shown in the Sri-Lankan study [13]. It could also be due to the timing of blood sampling relative to the onset of disease, not always defined. Another hypothesis about this variation in levels of IL-10 may be linked to genetic traits from the patients. Indeed, several studies mentioned polymorphisms in IL-10 and TNF genes as susceptibility factors to different diseases. Interestingly, a meta-analysis concluded that polymorphisms in the *il-10* gene are closely associated with ethnicity. Two polymorphic loci (rs1800871 and rs1800896) are risk factors in the Asian population [14]. In addition to socio-professional risks, we may speculate that the Melanesian population could harbor some of these polymorphisms. A subsequent clinical randomized trial could include the search for *tlr*, *il-10*, and *tnf* single nucleotide polymorphisms that could confer higher susceptibility to leptospirosis in the Melanesian population. However, all this does not concern directly our purpose of JHR. Therefore, we did not include this discussion in the manuscript.

**Editorial and Data Presentation Modifications?**

Reviewer #1: Including genotyping of infecting strains based on lfb-1 sequencing is potentially helpful. Although genotype did not seem to be linked to whether JHR occurred, it would be helpful to examine whether there is a link between genotype and clinical and/or laboratory severity. **Specifically, was there any correlation between genotype and the level of leptospiremia?**

Answer: Additional statistical analyses have been performed and are presented in 2 supplementary Tables (3 and 4). These did not reveal any significant differences in socio-demographic characteristics or risk factors across genotypes, except for exposure to river water: all patients infected with genotype I5 reported such exposure (8/8), compared to 4/7 for B1, 18/36 for I1, and 7/10 for I2. The distribution of genotypes also differed significantly by hospital of admission (p=0.00231), likely to reflect differences in environmental exposures in the corresponding catchment areas (sup table 3). This paragraph was added line 331.

Regarding clinical and laboratory severity, no significant association was found between genotype and the level of leptospiremia. a sentence was added line 338: no statistical difference of bacteremia was found between the different genotypes (Sup Tables 3 & 4). However, differences in median values were observed for certain biological parameters across genotypes, including platelet count, creatinine, ASAT, bilirubin, IL-10, and IL-1ß levels. It was added line 346.

**Summary and General Comments:**

Reviewer #1: This an innovative prospective study of 81 leptospirosis patients in New Caledonia compares the clinical and laboratory findings in leptospirosis patients with vs without Jarisch-Herxheimer reactions (JHR) following β-lactam antibiotic treatment. The novel findings of low baseline levels of pro-inflammatory cytokines and elevated IL-10 contribute greatly to our understanding of leptospirosis in that they challenge the cytokine storm model of leptospirosis.

**Will follow-up studies be performed to examine the longer-term consequences of JHR in leptospirosis, such as post-treatment fatigue, organ dysfunction, or persistent cytokine elevation?**

Answer: This is a very interesting suggestion, but it is not our priority. Our next goal is to help clinicians to avoid JHR by conducting randomized clinical trials to test and compare the effects of different classes of antibiotics, including bacteriostatic antibiotics such as azithromycin that are already used in some countries where JHR is not observed.

Answers to Reviewer 2’s **Questions**

**Methods**

Reviewer #2: This is a well design study; Thank you for this comment.

1. **Regarding to the definition of JHR, do we have the citation for this purpose?**

Answer: we started from Guerrier’s 2017 study (PMID: 28115662); but since the JRH definition was not detailed enough, clinicians from this study discussed together to define the presented JHR score.

2. The symptoms such as fever, chills, body ache and skin rash are often clinical manifestation of acute leptospirosis before treatment with an antibiotic, so worsening symptoms after treatment can be overlooked simply as signs of the underlying infection**. How do we differentiate the finding from the nature of clinical manifestations?**

Answer: we thank the reviewer for this question. The main difference is the timing and nature of JHR symptoms (as defined in Table 1) which occur suddenly within hours following antibiotic administration and resolved rapidly—typically within 6 hours—suggesting a transient, treatment-associated reaction rather than the natural course of the infection, whereas deterioration of patients may include other symptoms such as the typical renal and hepatic failure.

We added a sentence in the methods section line 243: “In fact, JHR symptoms occur suddenly within hours following antibiotic administration and resolved rapidly—typically within 6 hours—suggesting a transient, treatment-associated reaction rather than the natural course of the infection, whereas deterioration of patients may include other symptoms such as the typical renal and hepatic failure

Answer: Regarding the correlation between JHR and clinical worsening, both were evaluated in our study, although the assessment was ultimately based on the clinician’s judgment. The protocol specifically required investigators to report both the occurrence of JHR and any worsening of the patient’s condition, particularly at 24 hours post-admission (H24). The distinction between JHR and disease progression was based on the investigator’s clinical interpretation, considering the timing, symptom dynamics, and overall patient trajectory.

Among patients classified with JHR, 17 out of 35 (48.6%) experienced clinical worsening at H24. This proportion was 5 out of 10 (50%) for patients with possible JHR, and 10 out of 28 (35.7%) for patients without JHR. We were unable to demonstrate statistical evidence (p=0.268). This sentence was added in the result section line 448.

**Results**

Reviewer #2: 1. **What is the median day of fever at the time of enrolment? This timing may contribute to the level of cytokines at the presentation.**

Answer: we agree that this information is important, but this parameter was not recorded. In future studies, this information will be collected with precision. A sentence has been added in the discussion section in the paragraph about limitations of our study line 613: First, the delay between onset of first leptospirosis symptoms and antibiotic treatment was not recorded in the present study. It would have been an important parameter to consider confirming the above hypothesis.

**How to reduct this lead time bias?**

Answer: We didn’t find a proxy to evaluate the lead time bias with confidence.

**2. How do we explain the high rate of JHR in New Caledonia?**

Answer: There are several potential reasons for this phenomenon. The treatment method in New Caledonia primarily utilizes β-lactams, a class of bactericidal antibiotics, which may rapidly trigger the release of inflammatory cell wall components of leptospires. Additionally, some clinicians on the Northern Island, where care facilities are limited, are aware of leptospirosis-associated JHR and are more carefully monitoring the temperature than in other countries where patients enter hospitals with ICU, where there is better handling of serious complications and less attention to the patients changes in temperature or shivering.

**Conclusions**

Reviewer #2: I think the conclusion is a bit strong, for example conclusion as "This study confirms the stealth nature of leptospires, which do not induce inflammation but a potent anti-inflammatory response. It rejects the cytokine storm hypothesis often cited in leptospirosis and does not argue for the use of immunosuppressive drugs. When we look to see the results, there is also increase level of IL-6, and TNF following time points, not only IL-10.

Answer: We agree that the conclusion was strong, and we rewrote this paragraph to be less assertive:

Lines 45-49: This study confirms the stealth nature of leptospires, which induce a potent anti-inflammatory response rather than inflammation. It calls into question both the cytokine storm hypothesis, which is often cited in leptospirosis and the use of immunosuppressive drugs. The high incidence of JHR in New Caledonia suggests that the systematic use of ß-lactams as a first-line treatment should be reevaluated”

We also attenuated the sentence line 537: Importantly, the present results show that leptospirosis causes anti-inflammatory but almost no pro-inflammatory cytokine response in hospitalized patients.

**References**

1.	Guerrier G, D'Ortenzio E. The Jarisch-Herxheimer reaction in leptospirosis: a systematic review. PLoS One. 2013;8(3):e59266. Epub 20130326. doi: 10.1371/journal.pone.0059266. PubMed PMID: 23555644; PubMed Central PMCID: PMCPMC3608636.

2.	Tsuha S, Taniguchi T, Shiiki S, Narita M, Leung DT. Clinical characteristics of laboratory-confirmed leptospirosis in Okinawa, Japan, 1974-2015: high incidence of Jarisch-Herxheimer reaction. Trans R Soc Trop Med Hyg. 2016;110(9):558-65. Epub 20161015. doi: 10.1093/trstmh/trw061. PubMed PMID: 27744340.

3.	Peng RR, Wu J, Zhao W, Zhu L, Guan Z, Gu X, et al. An observational prospective study based on a large cohort of HIV-negative neurosyphilis patients with particular reference to the Jarisch-Herxheimer reaction. Eur J Clin Microbiol Infect Dis. 2024;43(6):1073-80. Epub 20240401. doi: 10.1007/s10096-024-04810-1. PubMed PMID: 38557924; PubMed Central PMCID: PMCPMC11178573.

4.	Dionne JA, Zhu C, Mejia-Galvis J, Workowski K, Batteiger TA, Dombrowski JC, et al. Jarisch-Herxheimer Reaction After Benzathine Penicillin G Treatment in Adults With Early Syphilis: Secondary Analysis of a Randomized Clinical Trial. JAMA Netw Open. 2025;8(2):e2459490. Epub 20250203. doi: 10.1001/jamanetworkopen.2024.59490. PubMed PMID: 39946129; PubMed Central PMCID: PMCPMC11826359.

5.	Butler T. The Jarisch-Herxheimer Reaction After Antibiotic Treatment of Spirochetal Infections: A Review of Recent Cases and Our Understanding of Pathogenesis. Am J Trop Med Hyg. 2017;96(1):46-52. Epub 20161024. doi: 10.4269/ajtmh.16-0434. PubMed PMID: 28077740; PubMed Central PMCID: PMCPMC5239707.

6.	Devlin AA, Halvorsen PJ, Miller JC, Laster SM. Il-10 deficient mice express IFN-gamma mRNA and clear Leptospira interrogans from their kidneys more rapidly than normal C57BL/6 mice. Immunobiology. 2017;222(5):768-77. Epub 20170210. doi: 10.1016/j.imbio.2017.02.004. PubMed PMID: 28237664.

7.	Xie X, Lv T, Wu D, Shi H, Zhang S, Xian X, et al. IL-10 Deficiency Protects Hamsters from Leptospira Infection. Infect Immun. 2022;90(2):e0058421. Epub 20211213. doi: 10.1128/IAI.00584-21. PubMed PMID: 34898251; PubMed Central PMCID: PMCPMC8852706.

8.	Papadopoulos S, Hardy D, Vernel-Pauillac F, Tichit M, Boneca IG, Werts C. Myocarditis and neutrophil-mediated vascular leakage but not cytokine storm associated with fatal murine leptospirosis. EBioMedicine. 2025;112:105571. Epub 20250130. doi: 10.1016/j.ebiom.2025.105571. PubMed PMID: 39889371; PubMed Central PMCID: PMCPMC11830356.

9.	Reis EA, Hagan JE, Ribeiro GS, Teixeira-Carvalho A, Martins-Filho OA, Montgomery RR, et al. Cytokine response signatures in disease progression and development of severe clinical outcomes for leptospirosis. PLoS Negl Trop Dis. 2013;7(9):e2457. Epub 20130919. doi: 10.1371/journal.pntd.0002457. PubMed PMID: 24069500; PubMed Central PMCID: PMCPMC3777885.

10.	Alikhani A, Mahmoodi K, Delavaryan L, Salehifar E, Rafiei AR, Zavare MS. Can cytokines response play a role in the treatment of fatal leptospirosis? Casp J Intern Med. 2022;13(2):343-8. doi: 10.22088/cjim.13.2.343. PubMed PMID: WOS:000792217100004.

11.	Wagenaar JF, Gasem MH, Goris MG, Leeflang M, Hartskeerl RA, van der Poll T, et al. Soluble ST2 levels are associated with bleeding in patients with severe Leptospirosis. PLoS Negl Trop Dis. 2009;3(6):e453. Epub 20090602. doi: 10.1371/journal.pntd.0000453. PubMed PMID: 19488407; PubMed Central PMCID: PMCPMC2684584.

12.	Senavirathna I, Rathish D, Agampodi S. Cytokine response in human leptospirosis with different clinical outcomes: a systematic review. BMC Infect Dis. 2020;20(1):268. Epub 20200407. doi: 10.1186/s12879-020-04986-9. PubMed PMID: 32264832; PubMed Central PMCID: PMCPMC7137275.

13.	Senavirathna I, Jayasundara D, Warnasekara J, Kappagoda C, Agampodi S. Levels of Cytokines in Leptospirosis Patients with Different Serovars and rfb Locus. J Interferon Cytokine Res. 2024;44(2):80-93. doi: 10.1089/jir.2023.0091. PubMed PMID: 38377491; PubMed Central PMCID: PMCPMC10880283.

14.	Wang S, Liang T, Zhang C. The relationship between expression level and gene polymorphism of inflammatory factors and sepsis risk. Sci Rep. 2025;15(1):6701. Epub 20250225. doi: 10.1038/s41598-025-90995-0. PubMed PMID: 40000789; PubMed Central PMCID: PMCPMC11861965.
